# A review of the species of Rhynchopsilopa Hendel from China (Diptera, Ephydridae)

**DOI:** 10.3897/zookeys.216.3224

**Published:** 2012-08-21

**Authors:** Junhua Zhang, Ding Yang, Wayne N. Mathis

**Affiliations:** 1 Institute of Plant Quarantine, Chinese Academy of Inspection and Quarantine, Beijing 100029, China (zjhcome@yahoo.com.cn); 2 Department of Entomology, China Agricultural University, Beijing 100193, China; 3 Department of Entomology, Smithsonian Institution, NHP 169, PO Box 37012, Washington, D. C. 20013-7012, USA

**Keywords:** Diptera, Ephydridae, *Rhynchopsilopa*, new species, China

## Abstract

Species of the shore-fly genus *Rhynchopsilopa* Hendel from China are reviewed. Four new species (*Rhynchopsilopa guangdongensis* **sp. n.**, *Rhynchopsilopa huangkengensis* **sp. n.**, *Rhynchopsilopa jinxiuensis* **sp. n.**, *Rhynchopsilopa shixingensis* **sp. n.**) and two previously known species, *Rhynchopsilopa longicornis* (Okada) and *Rhynchopsilopa magnicornis* Hendel, are described or redescribed. A key to the species hitherto known from China is presented.

## Introduction

Among shore flies, *Rhynchopsilopa* is apparently unique in having an association with ants (Farquharson 1921, Wirth 1968, Freidberg and Mathis 1985). Freidberg and Mathis (1985) demonstrated through choice experiments that this association, which may be obligate, is specific to workers of the genus *Crematogaster* Lund, with no apparent association with other ant genera. *Crematogaster* is an abundant, ecologically diverse genus of ants that is found worldwide and is easily recognized by its unusual, heart-shaped gaster. The adult flies are proctophiles on workers of *Crematogaster* and feed by injecting digestive liquids through the anus and into the abdomen of the ant prey. The fly then ingests the resultant slurry of partially digested liquids from the ant’s abdomen. We know nothing about the immature stages of *Rhynchopsilopa*, nor has a biological association with *Crematogaster* been documented for most of the species.

The unique and somewhat bizarre biology of *Rhynchopsilopa* is not the only feature that makes *Rhynchopsilopa* appealing to research. Adults of *Rhynchopsilopa* are relatively attractive in having a shiny habitus that is metallic dark blue to black in luster and color. Despite their striking appearance and exhibiting a unique biology, the basic systematics of the genus remains fragmentary and incomplete, with many undescribed species, especially from the Afrotropical Region (Freidberg personal communication).

The genus *Rhynchopsilopa* Hendel 1913 is one of 11 genera in the tribe Psilopini (subfamily Discomyzinae) and currently includes 20 species (Mathis and Zatwarnicki 1995). The genus is distinctive and is easily recognized by the long, pendant antennae; the short frons; the depressed face with a sharp epistoma; the long proboscis, and the convex thorax and abdomen (Wirth 1968). *Rhynchopsilopa* is only known from the Old World, and the Afrotropical Region has greatest species diversity, with 14 described species. One species, *Rhynchopsilopa nitidissima* Hendel, is known from the Palearctic Region, and five have been recorded from the Oriental Region. Of the five Oriental species, only two have been recorded from China (Cogan and Wirth 1977, Mathis and Zatwarnicki 1995): *Rhynchopsilopa longicornis* (Okada) and *Rhynchopsilopa magnicornis* Hendel. The purpose of this paper is to redescribe the species of *Rhynchopsilopa* that are known from China, and to describe four additional species as new to science. A key to the Chinese species is also provided.

## Material and methods

The descriptive terminology, with the exceptions noted in Mathis (1986) and Mathis and Zatwarnicki (1990a), follows that published in the Manual of Nearctic Diptera (McAlpine 1981). Because specimens are small, less than 2.50 mm in length, study and illustration of the male terminalia require use of a compound microscope. For most of the structures of the male terminalia, we follow the terminology that other workers in Ephydridae have used (see references in Mathis 1986 and Mathis and Zatwarnicki 1990a, 1990b). The species descriptions are composite and not based solely on holotypes.

### Two venational indices used in the descriptions are defined below

Costal vein index is the straight line distance between the apices of R_1_ and R_2+3_ (costal section II) divided by the distance between the apices of R_2+3_ and R_4+5_ (costal section III).

M vein ratio is the straight line distance apicad of crossvein dm-cu divided by the distance along M between crossvein dm-cu and r-m.

The holotypes and most paratypes are deposited in the Entomological Museum of the China Agricultural University (CAU), Beijing, some paratypes are also deposited in the National Museum of Natural History (USNM), Washington, D.C. We also studied specimens from the following museums: **BMNH** - The Natural Museum, London, England; **DEI** - Deutsches Entomologisches Institut, Müncheberg, Germany; and **ZMAN** - Instituut voor Taxonomische Zoologie, Zoologisch Museum, Universiteit van Amsterdam, Amsterdam, Netherlands. The following abbreviations are used for setae: acr = acrostichal, av = anteroventral, dc = dorsocentral, ia = intra-alar, npl = notopleural, oc = ocellar, orb = orbital, pd = posterodorsal, posts = postsutural, pres = presutural, psa = postalar, pv = posteroventral, sa = supra-alar, sc = scutellar, vt = vertical.

## Taxonomy

### Rhynchopsilopa

Hendel, 1913

http://species-id.net/wiki/Rhynchopsilopa

Rhynchopsilopa Hendel 1913: 96. Type species: *Rhynchopsilopa magnicornis*Hendel 1913, original designation. –Wirth 1968: 37–46 [review]. –Cogan and Wirth 1977: 330 [Oriental catalog]. –Freidberg and Mathis 1985: 13–20 [feeding habits].
Lissodrosophila Okada 1966: 45. Type species: *Lissodrosophila longicornis*Okada 1966, original designation. –Cogan and Wirth 1977: 330 [synonymy].

#### Diagnosis.

Small to moderately small shore flies, body length 1.7–2.8 mm; microtomentum generally sparse or lacking, cuticle appearing subshiny to shiny; mostly dark blue to black species.

Head in lateral view with antenna inserted at anterodorsal corner of head; frons conspicuously wider than long, often lenticular; a single, well-developed, proclinate fronto-orbital seta (sometimes an additional, distinctly shorter proclinate setula is present posteriad); reclinate seta and pseudopostocellar setae lacking or, in the latter case, very weakly developed; both medial and lateral vertical setae well developed; ocellar seta well developed, subequal in length to lateral vertical seta, proclinate, almost parallel; vertex convex; posterior ocelli situated immediately before convex vertex, ocelli forming an isosceles triangle. Antenna very elongate, pendant; scape exerted, oriented dorsally to anterodorsally; pedicel oriented anteroventrally, moderately elongate, lacking a prominent, well-developed dorsoapical seta; basal flagellomere pendant, very elongate, sometimes longer than face height; arista with 7–10 dorsal rays. Face depressed, mostly plain, lacking pits, transverse microrugosity or striae, bearing a sharp epistoma; a well-developed facial seta lacking; palpus whitish yellow to brown; proboscis elongate, longer than eye height, forming a well-sclerotized tube.

Thorax generally convex, dark blue to black, with microtomentum sparse to lacking; supra-alar seta absent; prescutellar acrostichal seta well developed; only posteriormost dorsocentral seta well developed; scutellum conspicuously wider than long, posterior margin broadly rounded, disc sparsely setulose; basal scutellar seta at most about 1/2 length or less than apical seta; anepisternum with 2 large setae. Wing mostly hyaline; crossveins not darkened; vein R_2+3_ usually extended to costal margin, lacking stump vein; R stem vein bare of setulae dorsally. Knob of haltere yellow to tan. Legs yellow to dark brown; forebasitarsus yellow to tan, only apical 1–2 tarsomeres dark brown.

Abdomen generally convex, bare of microtomentum, shiny, blackish; tergites 3–4 long, 5th tergite very short and lacking prominent, dorsally erect setae along posterior margin. Male terminalia: epandrium in posterior view as an inverted, rounded U (open ventrally), in lateral view generally elongate, usually thin to very thin, often slightly wider subventrally; cercus in posterior view thinly lunate to hemispherical; presurstylus, if present, short, no more than ½ length of postsurstylus, tapered to point ventroapically, apex bearing setulae, often greatly reduced or lacking; postsurstylus longer than wide, tapered to a ventral point, often with sinuous or curved margins; subepandrial plate usually bar-like, attenuate medially; pregonite bearing short setulae; aedeagus longer than wide, with sclerotized portion deeply bifurcate, appearing as 2 ventral extensions; phallapodeme long and narrow, in lateral view with a rod-like keel; hypandrium in lateral view moderately deep, pocket-like, or very shallow, nearly flat.

#### Key to species of *Rhynchopsilopa* from China

**Table:** 

1	Forefemur dark brown or brownish yellow	2
–	Forefemur yellow	4
2	Face metallic black with blue or brownish reflections; palpus brownish yellow or yellowish; forefemur with moderate pd and pv, at most as long as width of forefemur; mesonotum and abdomen with short and sparse setulae	3
–	Face white; palpus whitish yellow; forefemur with strong pd and pv, each long, about twice width of forefemur; mesonotum and abdomen with long and numerous setulae	*Rhynchopsilopa jinxiuensis* sp. n.
3	Palpus brownish yellow; forecoxa brown at extreme base; costal vein index 0.43, M vein index 2.0; costal section I of male not thickened	*Rhynchopsilopa huangkengensis* sp. n.
–	Palpus and forecoxa yellowish; costal vein index 0.33, M vein index 2.2; costal section I of male greatly thickened	*Rhynchopsilopa magnicornis* Hendel
4	Mid and hind tarsomeres 4–5 dark; hypandrium large, postsurstylus broad at apex, but pointed at extreme apex, gonite slender at base	*Rhynchopsilopa shixingensis* sp. n.
–	Mid and hind tarsomeres 5 dark; hypandrium small, postsurstylus tapering at apex, gonite short and thick	5
5	Body brownish yellow; face reddish orange; palpus brown; mid and hind femora yellow	*Rhynchopsilopa guangdongensis* sp. n.
–	Body black with blue reflections; face metallic black with blue reflections; palpus yellow; mid and hind femora dark brown	*Rhynchopsilopa longicornis* (Okada)

### Rhynchopsilopa guangdongensis sp. n.

urn:lsid:zoobank.org:act:7523AD49-FB44-4FFD-AA17-A0A42894E659

http://species-id.net/wiki/Rhynchopsilopa_guangdongensis

Figs 1–8

#### Diagnosis.

Body brownish yellow. Face subshiny, reddish orange; epistoma yellow; palpus brown, not stout at apex; arista with 8 dorsal rays. 1 pair of posts dc, sutural dc absent. Forecoxa yellowish, mid and hind coxae brownish yellow; femora yellow; tibia and tarsomeres 1–4 yellowish, tarsomere 5 dark. Forefemur with a row of pd and pv shorter than width of forefemur. Mesonotum and abdomen with short and sparse setulae. Costal vein index 0.45, M vein index 2.1. Male genitalia: epandrium narrow; hypandrium in ventral view hourglass-like, in lateral view shallow to nearly flat; postsurstylus tapered toward apex in lateral view; gonite/subepandrial plate shallowly sinuous, rod-like; phallapodeme vertically elongate, with short extended keel oriented more toward hypandrial attachment of phallapodeme.

#### Description.

Male. Body length 2.0–2.2 mm; wing length 2.3–2.4 mm.

Head subshiny, brownish red. Setulae and setae of head black; lateral vt as long as medial vt; 1 pair of strong oc; 1 pair of strong proclinate orb. Face subshiny, reddish orange; epistoma yellow; palpus brown. Gena with 1 strong seta. Arista with 8 dorsal rays.

**Figures 1–8.**
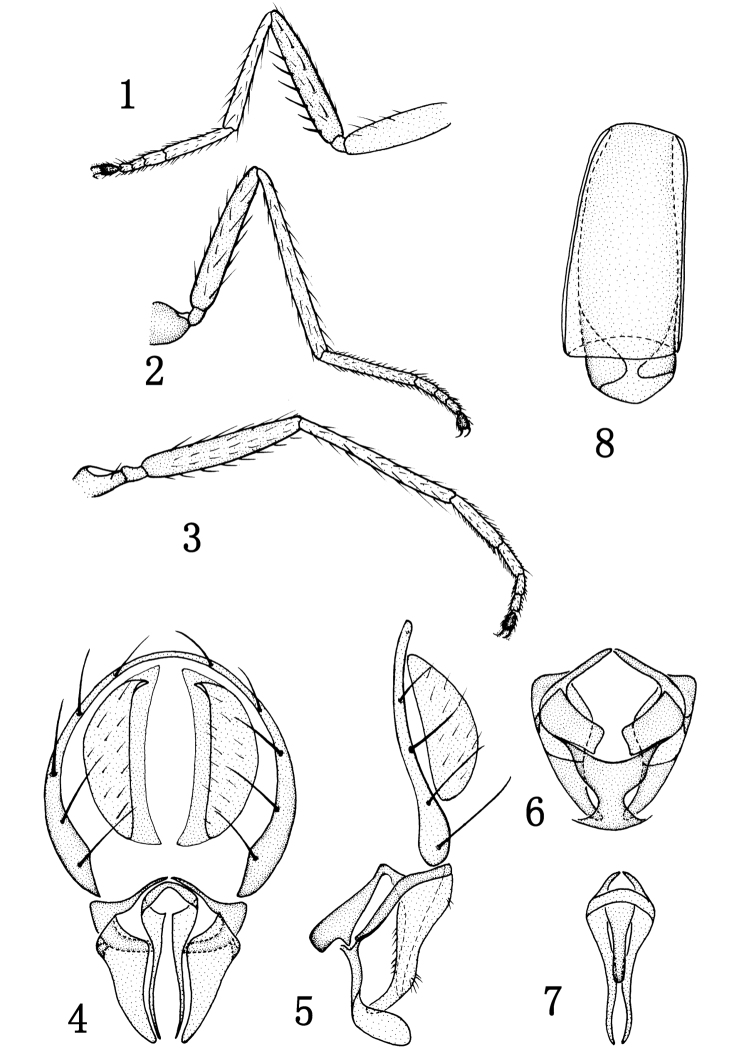
*Rhynchopsilopa guangdongensis* sp. n. (male) **1** foreleg **2** midleg **3** hindleg **4** terminalia (epandrium, cercus, postsurstyli, aedeagus), posterior view **5** terminalia (epandrium, cercus, postsurstyli, aedeagus, phallapodeme, gonite/subepandrial plate, hypandrium), lateral view **6** terminalia (surstyli, gonite/subepandrial plate and hypandrium), ventral view **7** aedeagus and phallapodeme, ventral view. (female) **8** Ventral receptacle.

Thorax subshiny, brown, with violet reflections; mesonotum dark brown, with short and sparse setulae; anepisternum and katepisternum brownish yellow. Thoracic setulae and setae black. 1 pair of posts dc, sutural dc absent; 2 rows of acr weak and short; posterior npl as long as anterior npl; anepisternum with 2 setae, length of ventral seta 2× that of dorsal seta; katepisternal seta weaker than ventral anepisternal seta; 1 weak sa, 1 strong ia; scutellum with 2 pairs of sc, apical sc stronger than lateral sc. Forecoxa yellowish, mid and hind coxae brownish yellow; femora yellow; tibiae and tarsomeres 1–4 yellowish, tarsomere 5 dark (Figs 1–3). Forefemur with rows of pd and pv shorter than width of forefemur. Costal vein index 0.45, M vein index 2.1. Wing and veins yellowish. Haltere white.

Abdomen subshiny, brownish yellow, bearing short and sparse setulae. Male genitalia (Figs 4–7): epandrium in lateral view (Fig. 5) very thin, bearing long setae on entire length along posterior margin; cercus in posterior view (Fig. 4) hemispherical; presurstylus greatly reduced; postsurstylus in posterior view (Fig. 4) broader basally, thereafter shallowly sinuous, tapered to point, in lateral view (Fig. 5) broad basally, evenly tapered at ventral margin, symmetrically sinuous at dorsal margin; aedeagus in posterior view (Figs 4, 7) narrowly elongate, more so than postsurstylus, slightly arched ventrally; phallapodeme in lateral view (Fig. 5) vertically elongate with short extended keel oriented more toward hypandrial attachment of phallapodeme; subepandrial plate in posterior view (Fig. 4) rod-like, shallowly curved, not attenuate medially; hypandrium in ventral view (Fig. 6) hourglass-like, in lateral view (Fig. 5) shallow to nearly flat.

Female. Body length 1.7–2.0 mm; wing length 2.3–2.5 mm. Similar to male. Female ventral receptacle as in Fig 8.

#### Specimens examined.

Holotype **♂**, Guangdong: Dapu, Fengxi National Nature Reserve, 28 Jul 2003, Xingyue Liu (CAU). Paratypes 1**♀**, same data as holotype (CAU); 1**♂**, 1**♀**, Guangdong: Dapu, Fengxi National Nature Reserve, 29 Jul 2003, Shuwen An (CAU & USNM); 1**♂**, Guangdong: Dapu, Fengxi National Nature Reserve, 30 Jul 2003, Xingyue Liu (CAU); 1**♀**, Guangxi: Luocheng, Jiuwanshan National Nature Reserve, Yuxi, 28 Jul 2003, Lili Zhang (USNM).

#### Distribution. 

China (Guangdong, Guangxi).

#### Etymology.

The species epithet is derived from the type locality, Guangdong.

#### Remarks.

This new species is similar to *Rhynchopsilopa pallipes* Wirth but may be distinguished from the latter by the reddish orange face, the costal vein index (0.45), the M vein index (2.1), and by the darkened 5th tarsomere. In *Rhynchopsilopa pallipes*, the face is yellow, the costal vein index is 0.59, the M vein index is 2.1, and all tarsi are yellow (Wirth 1968).

### Rhynchopsilopa huangkengensis sp. n.

urn:lsid:zoobank.org:act:504B5DEB-A780-4B96-BD4F-B0C356765374

http://species-id.net/wiki/Rhynchopsilopa_huangkengensis

Figs 9–16

#### Diagnosis.

Body shiny black, with some bluish reflections. Face black, with blue reflections; palpus brownish yellow, moderate at apex; arista with 7–8 dorsal rays. 1 pair of posts dc, sutural dc absent. Forecoxa brown at extreme base, yellow at apex, mid and hind coxae brown; femora dark brown; tibiae and tarsomeres 1–4 yellowish, tarsomere 5 brown. Forefemur with rows of pd and pv, each about as long as width of forefemur; mid femur with a row of av, which are shorter than width of mid femur. Mesonotum and abdomen with short and sparse setulae. Costal vein index 0.43, M vein index 2.0; first costal section of male not thickened. Male genitalia: epandrium very thin, only slightly expanded ventrally; hypandrium in ventral view hourglass-like, with rounded anterior margin, in lateral view shallow to nearly flat; postsurstylus wide at base and slender at apex in lateral view; gonite/subepandrial plate slender, sinuous; phallapodeme with long, extended keel.

#### Description.

Male body length: 1.9–2.1 mm; wing length: 2.4–2.6 mm.

Head shiny black, with blue reflections. Setulae and setae of head black. Lateral vt as long as medial vt; 1 pair of strong oc; 1 pair of proclinate orb. Face black, with blue reflections; epistoma brownish yellow; palpus brownish yellow, moderate at apex. Gena with 1 strong seta. Arista with 7–8 dorsal rays.

**Figures 9–16.**
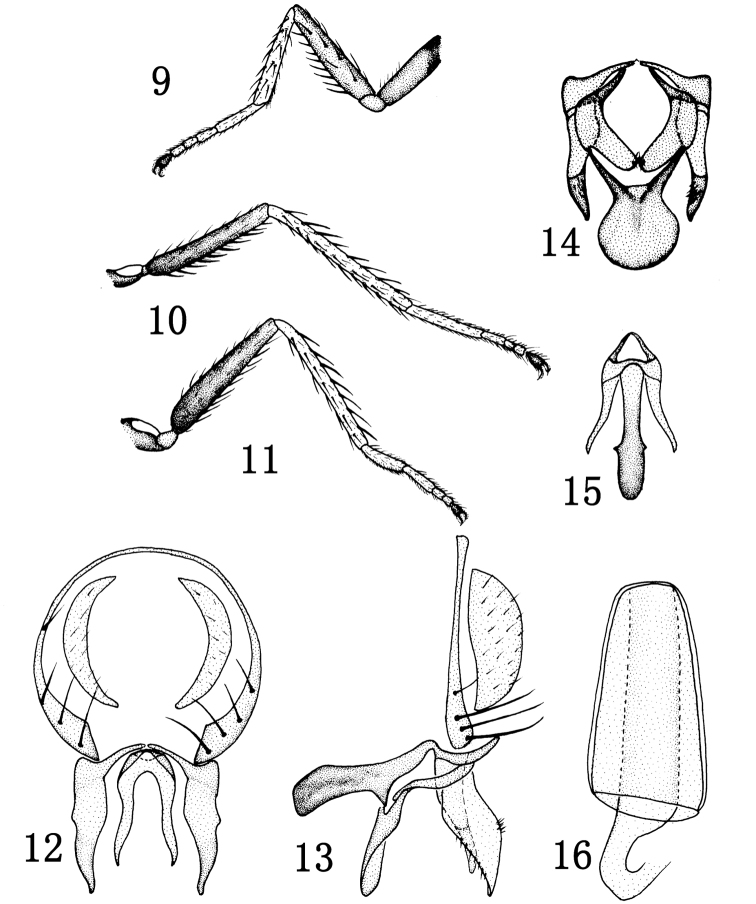
*Rhynchopsilopa huangkengensis* sp. n. (male) **9** foreleg **10** midleg **11** hindleg **12** terminalia (epandrium, cercus, postsurstyli, aedeagus), posterior view **13** terminalia (epandrium, cercus, postsurstyli, aedeagus, phallapodeme, gonite/subepandrial plate, hypandrium), lateral view **14** terminalia (presurstyli, postsurstyli, gonite/subepandrial plate, hypandrium), ventral view **15** aedeagus and phallapodeme, ventral view. (female) **16** Ventral receptacle.

Thorax shiny black, with blue reflections; mesonotum with short and sparse setulae. Thoracic setulae and setae black. 1 pair of posts dc, sutural dc absent; 2 rows of weak, short acr, posterior npl as long as anterior npl; katepisternal seta weaker than anepisternal seta; 1 weak sa, 1 strong ia; scutellum with 2 pairs of sc, apical sc stronger than lateral sc. Forecoxa brown at extreme base, yellow at apex, mid and hind coxae brown; femora dark brown; tibiae and tarsomeres 1–4 yellow, tarsomere 5 brown (Figs 9–11). Forefemur with rows of pd and pv, each nearly as long as width of forefemur; mid femur with a row of av, which are shorter than width of mid femur. Costal vein index 0.43, M vein index 2.0; first costal section of male not thickened. Wing brownish yellow, veins brown. Haltere white.

Abdomen shiny black, with blue reflections, bearing short and sparse setulae. Male genitalia (Figs 12–15): epandrium in posterior view (Fig. 12) very thin, bearing long setae on ventral half along posterior margin; cercus in posterior view (Fig. 12) narrowly lunate; presurstylus greatly reduced; postsurstylus in posterior view (Fig. 12) broader basally, thereafter shallowly sinuous with a shallow, lateral bump, tapered to ventral point, in lateral view (Fig. 13) more or less evenly broad on basal half, ventral half tapered to ventral point, more symmetrically angulate; aedeagus in posterior view (Figs 12, 15) narrowly elongate, more so than postsurstylus, slightly arched on ventral ¼; phallapodeme in lateral view (Fig. 13) transversely elongate, with long extended, more or less evenly thick keel; subepandrial plate in posterior view (Fig. 12) rod-like, shallowly curved; hypandrium in ventral view (Fig. 14) hourglass-like, with anterior margin rounded, in lateral view (Fig. 13) shallow to nearly flat.

Female. Body length: 2.4–2.8 mm; wing length: 2.7–2.8 mm. Similar to male. Female ventral receptacle as in Fig. 16.

#### Specimens examined.

Holotype **♂**, Fujian: Huangkeng, Aotou, 2 May 2004, Xingxue Liu (CAU). Paratypes 1**♀**, same data as holotype (CAU); 1**♂**, 2**♀♀**, Fujian: Huangkeng, Aotou, 1 May 2004, Dakang Zhou (CAU); 1**♂**, Fujian: Huangkeng, Aotou, 2 May 2004, Lili Zhang (CAU); 1**♂**, 3**♀♀**, Guangdong: Dapu, Fengxi National Nature Reserve, 29 Jul 2003, Shuwen An (CAU); 2**♂♂**, Guangdong: Dapu, Fengxi National Nature Reserve, 28 Jul 2003, Xingyue Liu (CAU); 1**♂**, Guangdong: Nanling National Nature Reserve, Qinshuigu, 25 Aug 2005, Junhua Zhang (CAU); 2**♂♂**, Guangdong: Nanling National Nature Reserve, Shumuyuan, 8 May 2004, Mengqing Wang (CAU); 5**♂♂**, 9**♀♀**, Guangdong: Nanling National Nature Reserve, Shumuyuan, 8 May 2004, Yang Ding (CAU); 3**♂♂**, Guangdong: Shaoguan, Chebaling National Nature Reserve, 12 Jul 2003, Shuwen An (CAU); 9**♂♂**, 7**♀♀**, Guangxi: Jinxiu, Dayaoshan National Nature Reserve, Fenzhancun, 23 Jul 2005, Yajun Zhu (CAU & USNM); 3**♂♂**, 2**♀♀**, Guangxi: Jinxiu, Dayaoshan National Nature Reserve, Hekou, 31 Jul 2005, Yajun Zhu (CAU); 5**♂♂**, 3**♀♀**, Guangxi: Jinxiu, Dayaoshan National Nature Reserve, Luoxiangcun, 28 Jul 2005, Yajun Zhu (CAU & USNM); 2**♂♂**, Guizhou: Libo, Yaolancun, 12 Jun 2005, Junhua Zhang (CAU); 1**♀**, Fujian: Huangkeng, Aotou, 2 May 2004, Junhua Zhang (CAU); 2**♀♀**, Guangdong: Zengcheng, Nankunshan, 15 Jul 2003, Xingyue Liu (CAU).

#### Distribution.

China (Fujian, Guangdong, Guangxi, Guizhou).

#### Etymology.

The species epithet is derived from the type locality, Huangkeng.

#### Remarks.

This new species is similar to *Rhynchopsilopa magnicornis* Hendel, but may be distinguished from the latter by the following characters: palpus brownish, forecoxa with a brown base, costal vein index 0.43, M vein index 2.0, and costal section I of the male not thickened. In *Rhynchopsilopa fuscipennis* Wirth, the palpus is yellowish, the forecoxa is yellowish, the costal vein index is 0.50, the M vein index is 2.2; and the costal section I of the male is thickened (Wirth 1968).

### Rhynchopsilopa jinxiuensis sp. n.

urn:lsid:zoobank.org:act:B2503CA3-0586-4EA1-8B9D-BE5883B33E2C

http://species-id.net/wiki/Rhynchopsilopa_jinxiuensis

Figs 17–24

#### Diagnosis.

Body shiny black, with blue reflections. Face white; palpus white, stout at apex; arista with ten dorsal rays. 1 pair of posts dc, sutural dc absent. Forecoxa yellowish, mid and hind coxae brownish yellow; femora dark brown; tibiae and tarsomeres 1–4 yellowish, tarsomere 5 dark. Forefemur with strong pd and pv, each long, about twice width of forefemur; mid femur with a row of strong av. Mesonotum and abdomen with long and numerous setulae. Costal vein index 0.30, M vein index 2.0. Male genitalia: epandrium moderately wide, especially subventrally; hypandrium in ventral view anchor-like, in lateral view with narrow base and expanded anterior extension; postsurstylus with ventral half bearing a long, narrow process extended from anteroventral angle of basal portion, forming a long, curved anterior margin; gonite thick at base and slender at apex in ventral view; phallapodeme with arched base, extended keel narrow, elongate, width of keel somewhat uniform.

#### Description.

Male body length: 2.1–2.4 mm; wing length: 2.8–3.0 mm.

Head shiny black, with blue reflections. Setulae and setae of head black. Lateral vt as long as medial vt; 1 pair of strong oc; 1 pair of lateroclinate orb. Face, epistoma, and palpus white, the latter stout at apex. Gena with 1 strong seta. Arista with 10 dorsal rays.

**Figures 17–24.**
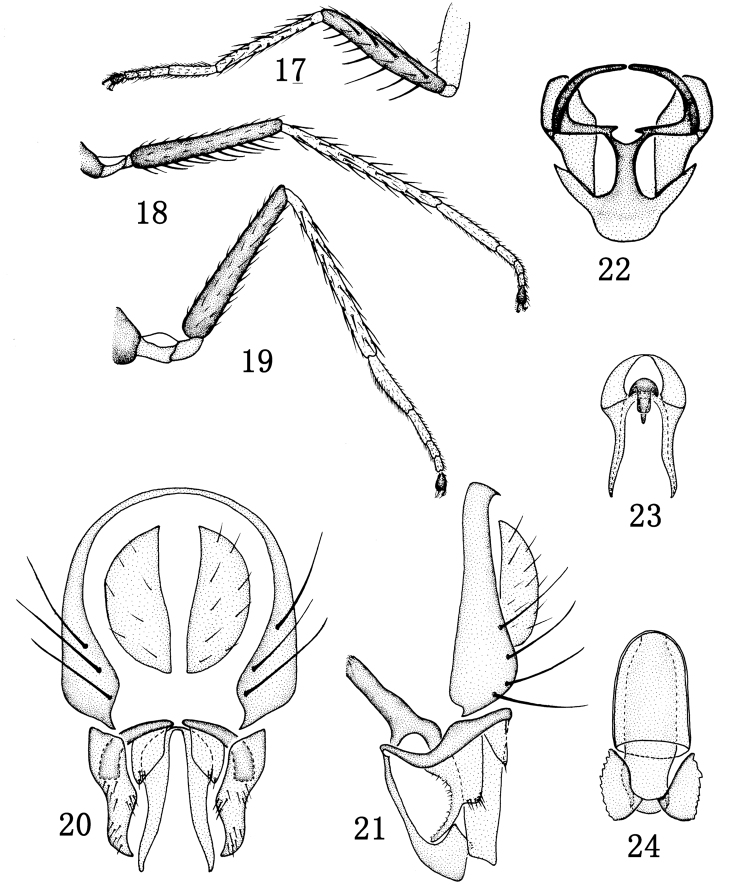
*Rhynchopsilopa jinxiuensis* sp. n. (male) **17** foreleg **18** midleg **19** hindleg **20** terminalia (epandrium, cercus, presurstyli, postsurstyli, aedeagus), posterior view **21** terminalia (epandrium, cercus, presurstylus, postsurstylis, aedeagus, phallapodeme, gonite/subepandrial plate, hypandrium), lateral view **22** terminalia (surstyli, gonite/subepandrial plate, hypandrium), ventral view **23** aedeagus and phallapodeme, ventral view. (female) **24** Ventral receptacle.

Thorax shiny black, with blue reflections; mesonotum with long and numerous setulae. Thoracic setulae and setae black. 1 pair of posts dc, sutural dc absent; 2 rows of long and numerous acr; posterior npl as long as anterior npl; katepisternal seta weaker than anepisternal seta; 1 weak sa, 1 strong ia; scutellum with 2 pairs of sc, apical sc stronger than lateral sc. Forecoxa yellowish, mid and hind coxae brownish yellow; femora dark brown; tibiae and tarsomeres 1–4 yellowish, tarsomere 5 dark (Figs 17–19). Forefemur with strong pd and pv, each long, about 2× width of forefemur; mid femur with a row of strong av. Costal vein index 0.30, M vein index 2.0. Wing brownish yellow, veins brown. Haltere white.

Abdomen shiny black, with blue reflections. Abdomen with long and numerous setulae. Male genitalia (Figs 20–23): epandrium in lateral view (Fig. 21) moderately thin, bearing long setae on ventral portion, along posterior margin; cercus in posterior view (Fig. 20) hemispherical; presurstylus small, in posterior view parallelogram-like (Fig. 20), with acute angle ventrad, less than ½ length of postsurstylus; postsurstylus in posterior view (Fig. 20) broader basally, thereafter tapered to ventral point, concave curve at outer margin and convex curve at inner margin, bearing setulae, in lateral view (Fig. 21) with basal half roughly triangular, slightly tapered ventrally, ventral half bearing a long, narrow process extended from anteroventral angle of basal portion, forming a long, curved anterior margin; aedeagus in posterior view (Figs 20, 23) narrowly elongate, more so than postsurstylus, slightly splayed latero-ventrally; phallapodeme in lateral view (Fig. 21) with arched base, extended keel narrow, elongate, width of keel somewhat uniform; subepandrial plate in posterior view (Fig. 20) rod-like, curved, attenuate medially; hypandrium in ventral view (Fig. 22) anchor-like, in lateral view (Fig. 21) with narrow base and expanded anterior extension.

Female. Body length: 2.3–2.4 mm; wing length: 2.8–3.0 mm. Similar to male. Female ventral receptacle as in Fig. 24.

#### Specimens examined.

Holotype **♂**, Guangxi: Jinxiu, Dayaoshan National Nature Reserve, Luoxiangcun, 28 Jul 2005, Yajun Zhu (CAU). Paratypes 25 **♂♂**, 1**♀**, same data as holotype (CAU & USNM); 1**♂**, 1**♀**, Guangdong: Dapu, Fengxi National Nature Reserve, 29 Jul 2003, Shuwen An (CAU); 2 **♂♂**, Guangxi: Jinxiu, Dayaoshan National Nature Reserve, Hekou, 31 Jul 2005, Yajun Zhu (CAU).

#### Distribution.

China (Guangdong, Guangxi).

#### Etymology.

The species epithet is derived from the type locality, Jinxiu.

#### Remarks.

This new species is similar to *Rhynchopsilopa fuscipennis* Wirth, from which it may be distinguished by having 10 dorsal aristal rays, costal vein index of 0.30, and M vein index of 2.0. In *Rhynchopsilopa fuscipennis* Wirth, the arista has 7 dorsal rays, the costal vein index is 0.50, and the M vein index is 1.8 (Wirth 1968).

### Rhynchopsilopa longicornis

(Okada)

http://species-id.net/wiki/Rhynchopsilopa_longicornis

Figs 25–32

Lissodrosophila longicornis Okada 1966: 45 [Nepal. Taplejung District, below Sangu; HT ♂, BMNH].
Rhynchopsilopa longicornis . –Cogan and Wirth 1977: 330 [Oriental catalog; generic combination]. –Mathis and Zatwarnicki 1995: 48 [world catalog].
Rhynchopsilopa coei Wirth 1968: 41 [Nepal. Taplejung: North of Sangu (5000 ft); HT ♀, BMNH]. –Cogan and Wirth 1977: 330 [synonymy].

#### Diagnosis.

Face shiny black, with blue reflections; palpus yellow; epistoma brownish yellow; arista with 9 dorsal rays. 1 pair of posts dc, sutural dc absent. Forecoxa brown at extreme base, mid and hind coxae brown; forefemur yellow, mid and hind femora dark brown at base; tibiae and tarsomeres 1–4 yellowish, tarsomere 5 dark. Forefemur with pd and pv, about as long as width of forefemur; mid femur with a row of av. Mesonotum and abdomen with short and sparse setulae. Costal vein index 0.50, M vein index 2.2. Male genitalia: epandrium narrow; hypandrium in ventral view hourglass-like, with anterior margin shallowly rounded, in lateral view shallow to nearly flat; postsurstylus tapered toward apex in lateral view; gonite/subepandrial plate slightly thick; phallapodeme with process at middle in lateral view.

#### Description.

Male body length: 1.8–2.0 mm; wing length: 2.1–2.4 mm.

Head shiny black, with blue reflections. Setulae and setae of head black. Lateral vt as long as medial vt; 1 pair of strong oc; 1 pair of proclinate orb. Face shiny black, with blue reflections; palpus yellow, stout at apex; epistoma brownish yellow. Gena with 1 strong seta. Arista with 9 dorsal rays.

**Figures 25–32.**
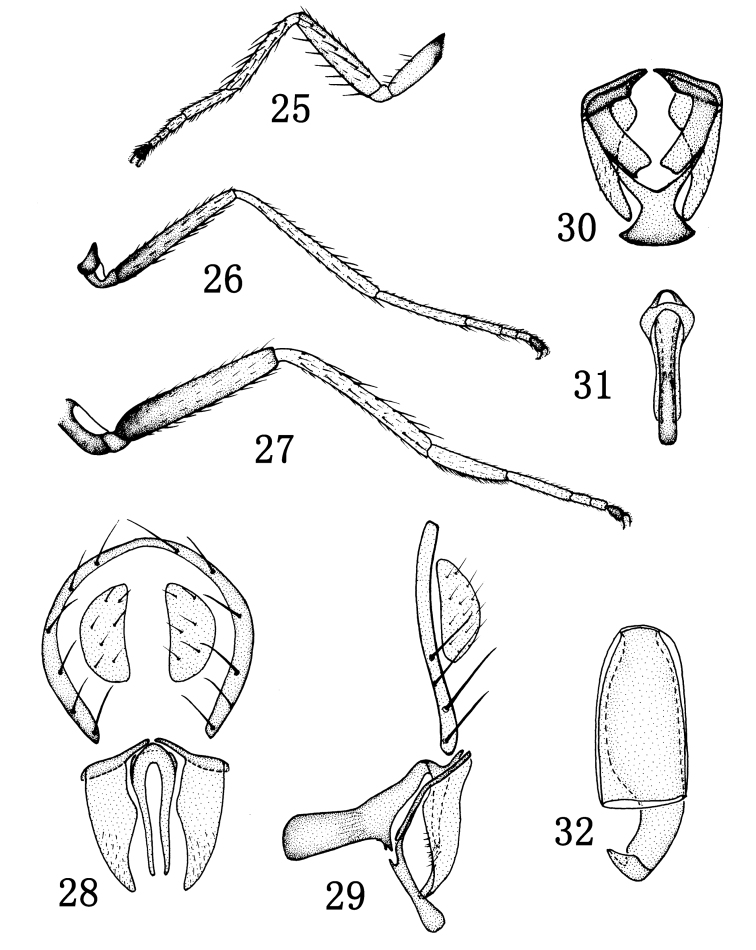
*Rhynchopsilopa longicornis* (Okada) (male) **25** foreleg **26** midleg **27** hindleg **28** terminalia (epandrium, cercus, postsurstyli, aedeagus), posterior view **29** terminalia (epandrium, cercus, postsurstylus, aedeagus, phallapodeme, gonite/subepandrial plate, hypandrium), lateral view **30** terminalia (presurstyli, postsurstyli, gonite/subepandrial plate, hypandrium), ventral view **31** aedeagus and phallapodeme, ventral view. (female) **32** Ventral receptacle.

Thorax shiny black, with blue reflections; mesonotum with short and sparse setulae. Thoracic setulae and setae black. 1 pair of posts dc, sutural dc absent; 2 rows of weak and short acr; posterior npl as long as anterior npl; anepisternum with 2 strong setae; 1 strong katepisternal seta, weaker than anepisternal seta; 1 weak sa, 1 strong ia seta; scutellum with 2 pairs of sc, apical sc stronger than lateral sc. Forecoxa yellow, with brown extreme base, mid and hind coxae brown; forefemur yellow, mid and hind femora dark brown, with yellow apex; tibiae and tarsomeres 1–4 yellowish, tarsomere 5 dark (Figs 25–27). Forefemur with rows of pd and pv, about as long as width of forefemur; mid femur with a row of av. Costal vein index 0.50, M vein index 2.2. Wing brownish yellow, veins brown. Haltere white.

Abdomen shiny black, with blue reflections. Abdomen with long and numerous setulae. Male genitalia (Figs 28–31): epandrium in lateral view (Fig. 29) very thin, bearing long setae on entire length along posterior margin; cercus in posterior view (Fig. 28) hemispherical; presurstylus greatly reduced; postsurstylus in posterior view (Fig. 28) evenly broad on basal half, thereafter tapered to ventral point, medial surface of ventral half shallowly concave, in lateral view (Fig. 29) more or less evenly tapered and smoothly sinuous from broad base to pointed apex; aedeagus in posterior view (Figs 28, 31) narrowly elongate, more so than postsurstylus, ventral extensions nearly straight; phallapodeme in lateral view (Fig. 29) transversely elongate with long extended, more or less evenly thick keel; subepandrial plate in ventral view (Fig. 30) slightly thick; hypandrium in ventral view (Fig. 30) hourglass-like, with anterior margin shallowly rounded, in lateral view (Fig. 29) shallow to nearly flat.

Female. Body length: 1.9–2.0 mm; wing length: 2.3–2.4 mm. Similar to male. Female ventral receptacle as in Fig. 32.

#### Specimens examined.

1**♂**, Guangdong: Dapu, Fengxi National Nature Reserve, 29 Jul 2003, Shuwen An (CAU); 4**♂♂**, 2**♀♀**, Guangdong: Dapu, Fengxi National Nature Reserve, 28 Jul 2003, Xingyue Liu (CAU); 1**♂**, Guangdong: Shixing, Chebaling National Nature Reserve, 10 Jul 2003, Xingyue Liu (CAU); 4**♂♂**, Guangdong: Wuhua, Qimuzhang, 31 Jul 2003, Shuwen An (CAU); 1**♂**, Guangxi: Luocheng, Jiuwanshanyuxi, 28 Jul 2003, Lili Zhang (CAU); 1**♀**, Fujian: Huangkeng, Aotou, 2 May 2004, Xingyue Liu (CAU); 2**♀♀**, Guangdong: Dapuxian, Fengxi National Nature Reserve, 30 Jul 2003, Xingyue Liu (CAU).

#### Distribution.

China (Fujian, Guangdong, Guangxi); Nepal.

### Rhynchopsilopa magnicornis

Hendel

http://species-id.net/wiki/Rhynchopsilopa_magnicornis

Figs 33–39

Rhynchopsilopa magnicornis Hendel 1913: 96 [Taiwan. Kankau, Paroe, N Paiwan District; ST ♂ & ♀, DEI]. –Cogan and Wirth 1977: 330 [Oriental catalog]. –Mathis and Zatwarnicki 1995: 48 [world catalog].
Rhynchopsilopa rugosiscutata de Meijere 1916: 267 [Indonesia. Java: “G. Ungaran”; HT ♂, ZMA]. –Wirth 1968: 43 [synonymy].

#### Diagnosis.

Face brownish, epistome yellowish; palpus yellowish, short, distally stout; mesonotum metallic bluish violet, with sparse squamose pubescence; sutural dc absent; legs dark brown, forecoxa, tibiae, extreme apices of femora, and tarsomeres 1–4 yellowish; wing slightly brownish; costal vein index 0.33, M vein index 2.2; haltere whitish (Wirth 1968).

#### Description.

Male body length: 1.7–1.8 mm; wing length: 2.8–3.0 mm.

Head shiny black, with blue reflections. Setulae and setae of head black. Lateral vt as long as medial vt; 1 pair of strong oc; 1 pair of proclinate orb. Face and palpus yellow, the latter stout at apex; epistoma yellow. Gena with 1 strong seta. Arista with 8–9 dorsal rays.

**Figures 33–39.**
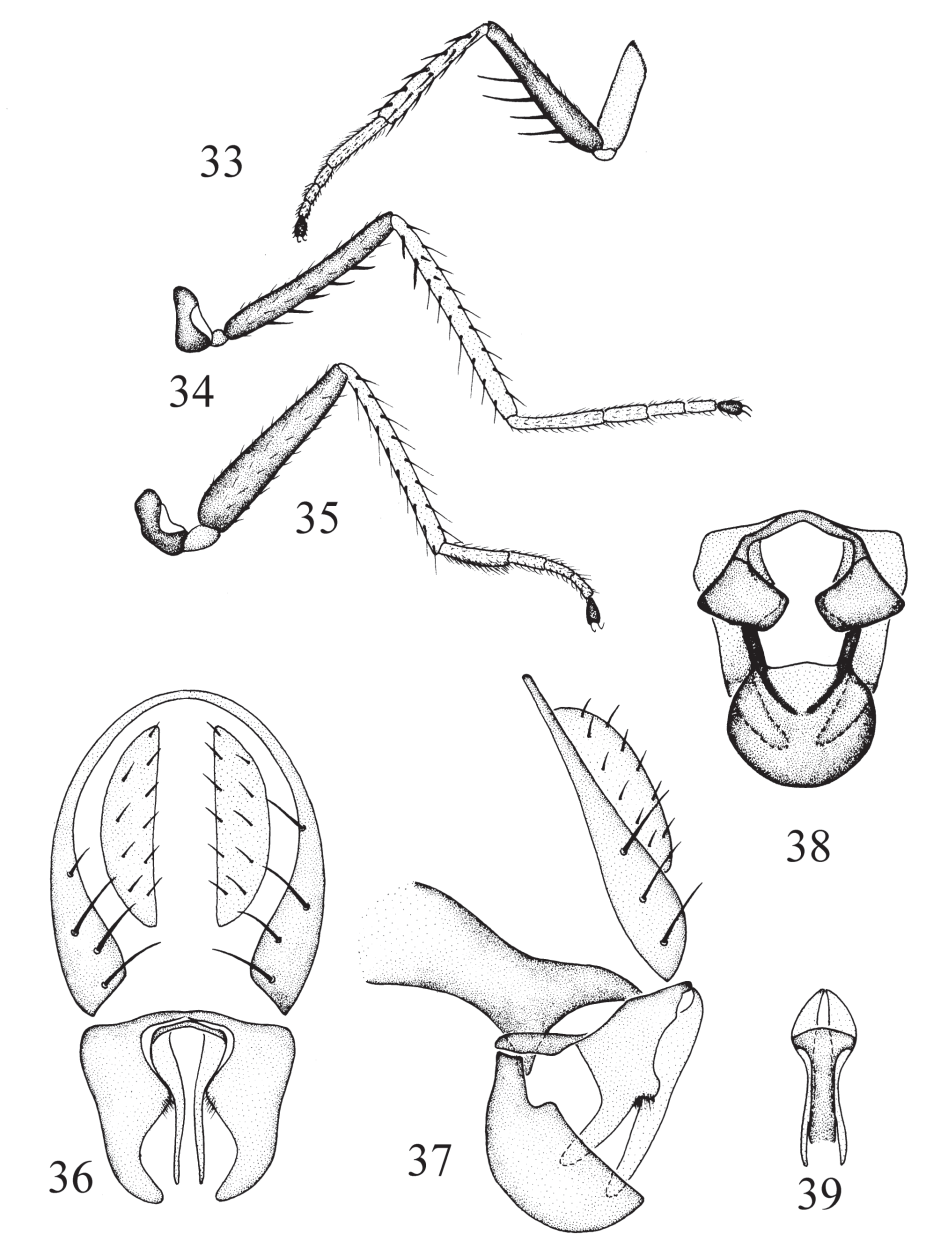
*Rhynchopsilopa magnicornis* Hendel(male) **33** foreleg **34** midleg **35** hindleg **36** terminalia (epandrium, cercus, postsurstyli, aedeagus), posterior view **37** terminalia (epandrium, cercus, postsurstylus, aedeagus, phallapodeme, gonite/subepandrial plate, hypandrium), lateral view **38** terminalia (presurstyli, postsurstyli, gonite/subepandrial plate, hypandrium), ventral view **39** aedeagus and phallapodeme, ventral view.

Thorax shiny black, with blue reflections; mesonotum with long and numerous setulae. Thoracic setulae and setae black. 1 pair of posts dc, sutural dc absent; 2 rows of acr long and numerous; posterior npl as long as anterior npl; katepisternal seta weaker than anepisternal seta; 1 weak sa, 1 strong ia; scutellum with 2 pairs of sc, apical sc stronger than lateral sc. Forecoxa yellowish, mid and hind coxae brownish yellow; femora dark brown; tibiae and tarsomeres 1–4 yellowish, tarsomere 5 dark (Figs 33–35). Forefemur with strong pv, about two times longer than width of forefemur; mid femur with a row of strong av. Costal vein index 0.33, M vein index 2.2. Wing brownish yellow, veins brown. Haltere yellow.

Abdomen shiny black, with blue reflections. Abdomen with long and numerous setulae. Male genitalia (Figs 36–39): epandrium in lateral view (Fig. 37) slightly wide, bearing long setae on ventral 2/3 along posterior margin; cercus in posterior view (Fig. 36) narrowly hemispherical; presurstylus greatly reduced; postsurstylus in posterior view (Fig. 36) robust, broader basally, thereafter unevenly tapered to pointed apex, medial margin deeply sinuous, in lateral view (Fig. 37) with basal half roughly triangular, slightly tapered ventrally, ventral half bearing a long, narrow process extended from anteroventral angle of basal portion, forming a long, slightly curved process from ventroanterior margin of basal portion, with a posterior knob at juncture of basal and ventral portions along porterior margin; aedeagus in posterior view (Figs 36, 39) narrowly elongate, more so than postsurstylus, ventrally extended process nearly straight; phallapodeme in lateral view (Fig. 37) transversely elongate with long extended, more or less evenly thick keel; subepandrial plate in ventral view (Fig. 38) subquadrangular; hypandrium in ventral view (Fig. 38) hourglass-like, with anterior margin broadly rounded, in lateral view (Fig. 37) deeply pocket-like, bowl shaped.

#### Specimens examined.

3**♂♂**, India: Meghalaga Nongph-Forest, 25–28 Apr 1980, A. Freidberg (CAU).

#### Distribution.

China (Taiwan), India, Indonesia (Java, Sumatra), Malaysia, Philippines (Mindanao, Tawi Tawi), Thailand.

### Rhynchopsilopa shixingensis sp. n.

urn:lsid:zoobank.org:act:0B787DBA-9ECC-4E25-9252-732941F150D7

http://species-id.net/wiki/Rhynchopsilopa_shixingensis

Figs 40–47

#### Diagnosis.

Body shiny black, with blue reflections. Face reddish brown; palpus yellow, not stout at apex; arista with 8–9 dorsal rays. 1 pair of posts dc, sutural dc absent. Forecoxa yellow, with brown extreme base, mid and hind coxae brownish yellow; femora and tibiae yellow; foretarsomere 5 brown, mid and hind tarsomeres 4 and 5 brown, other yellow. Forefemur with rows of strong pd and pv, longer than width of forefemur. Mesonotum and abdomen with short and sparse setulae. Costal vein index 0.45, M vein index 2.3. Male genitalia: epandrium narrow; hypandrium large, round in ventral view; postsurstylus broadened at apex, but pointed at extreme apex, gonite/subepandrial plate slender at base; phallapodeme with process at base in lateral view.

#### Description.

Male body length: 1.9–2.1 mm; wing length: 2.4–2.6 mm.

Head shiny black, with blue reflections. Setulae and setae of head black. Lateral vt as long as medial vt; 1 pair of strong oc; 1 pair of proclinate orb. Face reddish brown; epistoma and palpus yellow, the latter stout at apex. Gena with 1 strong seta. Arista with 8–9 dorsal rays.

**Figures 40–47.**
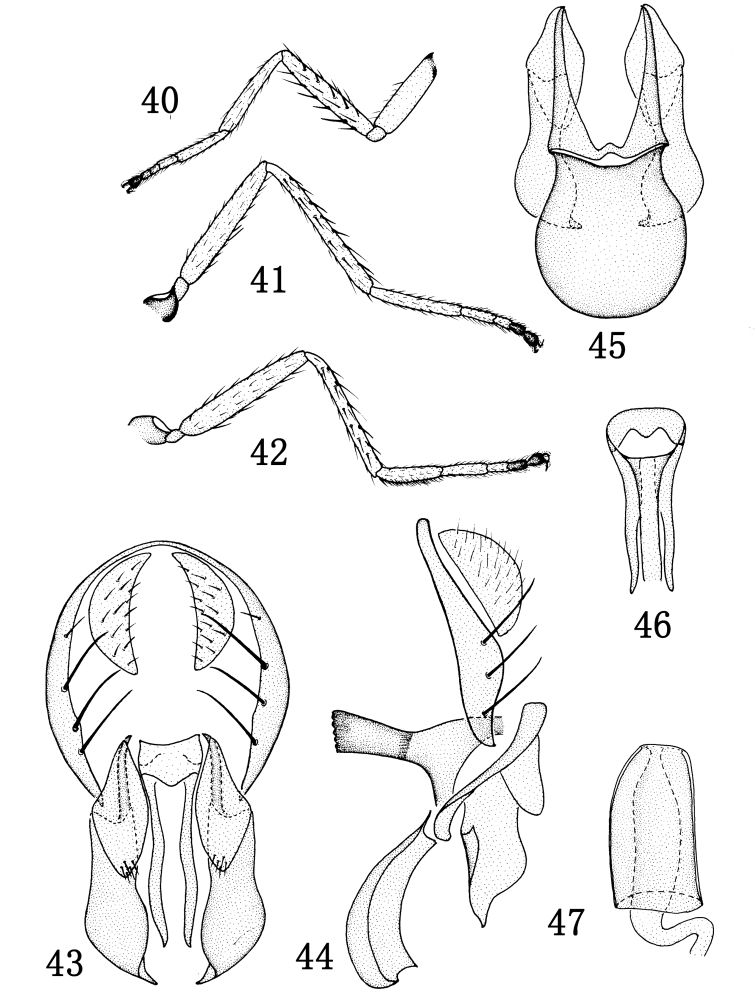
*Rhynchopsilopa shixingensis* sp. n. (male) **40** foreleg **41** midleg **42** hindleg **43** terminalia (epandrium, cercus, presurstyli, postsurstyli, aedeagus), posterior view **44** terminalia (epandrium, cercus, presurstylus, postsurstylus, aedeagus, phallapodeme, gonite/subepandrial plate, hypandrium), lateral view **45** terminalia (presurstyli, postsurstyli, gonite/subepandrial plate, hypandrium), ventral view **46** aedeagus and phallapodeme, ventral view. (female) **47** ventral receptacle.

Thorax shiny black, with blue reflections; mesonotum with short and sparse setulae. Thoracic setulae and setae black. 1 pair of posts dc, sutural dc absent; 2 rows of weak and short acr, posterior npl as long as anterior npl; katepisternal seta weaker than anepisternal seta; 1 weak sa, 1 strong ia; scutellum with 2 pairs of sc, apical sc stronger than lateral sc. Forecoxa yellow, with brown extreme base, mid and hind coxae brownish yellow; femora and tibiae yellow; foretarsomere 5 brown, mid and hind tarsomeres 4 and 5 brown, other yellow (Figs 40–42). Forefemur with rows of strong pd and pv, each longer than width of forefemur. Costal vein index 0.45, M vein index 2.3. Wing yellowish, veins yellow. Haltere white.

Abdomen shiny black, with blue reflections. Abdomen with short and sparse setulae. Male genitalia (Figs 43–46): epandrium in posterior view (Fig. 43) moderately thin, bearing long setae on ventral 2/3, along posterior margin; cercus in posterior view (Fig. 43) relatively short, lunate; presurstylus small, in posterior view parallelogram-like (Fig. 43), with acute angle ventrad, less than ½ length of postsurstylus; postsurstylus in posterior view (Fig. 43) becoming broader ventrally, sinuous to pointed, ventral apex, in lateral view (Fig. 44) with basal 2/3 roughly rectangular, abruptly tapered ventrally; aedeagus in posterior view (Figs 43, 46) narrowly elongate, more so than postsurstylus, ventrally extended processes shallowly sinuous; phallapodeme in lateral view (Fig. 44) with broad base, extended keel narrow, elongate, width of keel somewhat uniform; gonite/subepandrial plate in lateral view (Fig. 44) rod-like, sinuous; hypandrium in ventral view (Fig. 45) bulbous, anterior margin deeply rounded, in lateral view (Fig. 44) with narrow base and expanded anterior extension shallowly curved, moderately shallow.

Female. Body length: 2.4–2.80 mm; wing length: 2.70–2.80 mm. Similar to male. Female ventral receptacle as in Fig. 47.

#### Specimens examined.

Holotype **♂**, Guangdong: Shixingxian, Chebaling National Nature Reserve, 10 Jul 2003, Xingxue Liu (CAU). Paratypes 1**♂**, 1**♀**, same data as holotype (CAU); 1**♂**, Fujian: Huangkengxian, Aotou, 1 May 2004, Dakang Zhou (USNM); 1**♀**, Fujian: Huangkengxian, Aotou, 1 May 2004, Xingyue Liu (USNM); 1**♀**, Fujian: Huangkengxian, Aotou, 2 May 2004, Yajun Zhu (CAU).

#### Distribution.

China (Fujian, Guangdong).

#### Etymology.

The species epithet is derived from the type locality, Shixing.

#### Remarks.

This new species is similar to *Rhynchopsilopa magnicornis* Hendel, but may be distinguished from the latter by having a yellow palpus, the extreme base of the forecoxa brown, the costal vein index of 0.45, and the M vein index of 2.0. In *Rhynchopsilopa fuscipennis* Wirth, the palpus and forecoxa are yellowish, the costal vein index is 0.5, and the M vein index is 2.2 (Wirth 1968).

## Supplementary Material

XML Treatment for Rhynchopsilopa

XML Treatment for Rhynchopsilopa guangdongensis

XML Treatment for Rhynchopsilopa huangkengensis

XML Treatment for Rhynchopsilopa jinxiuensis

XML Treatment for Rhynchopsilopa longicornis

XML Treatment for Rhynchopsilopa magnicornis

XML Treatment for Rhynchopsilopa shixingensis
